# Paracrine effects of CCN3 from non-cancerous hepatic cells increase signaling and progression of hepatocellular carcinoma

**DOI:** 10.1186/s12885-019-5603-7

**Published:** 2019-04-27

**Authors:** Weimin Li, Xia Liao, Pengbo Ning, Yu Cao, Mei Zhang, Yang Bu, Jun Lv, Qingan Jia

**Affiliations:** 1 Department of Nutrition, First Affiliated Hospital of Xi’an Jiaotong University, Xi’an, 710061 China; 2 School of Life Science and Technology, Xidian University, Xi’an, Shaanxi China; 3 Department of pediatrics, First Affiliated Hospital of Xi’an Jiaotong University, Xi’an, 710061 China; 4 Department of Hepatobiliary Surgery, First Affiliated Hospital of Xi’an Jiaotong University, 277 West Yanta Road, Xi’an, 710061 China; 5 Department of Hepatobiliary Surgery, General Hospital, Ningxia Medical University, Yinchuan, 750001 China; 6 Clinical Research Center of Xi’an Jiaotong University, Xi’an, 710061 China

**Keywords:** Hepatocellular carcinoma, CCN3, Hepatic cell, Cirrhosis

## Abstract

**Background:**

The liver microenvironment plays a key role in the progression and metastasis of hepatocellular carcinoma (HCC). Gene expression profiling of non-cancerous hepatic tissues obtained from patients with metastatic HCC exhibit a unique immune response signature, including upregulation of CCN3. However, the role of CCN3 secreted from non-cancerous hepatic tissues in the progression of HCC remains unclear.

**Methods:**

Using tissue microarrays, we examined CCN3 in non-cancerous hepatic tissues of patients with HCC and correlated expression with clinical and pathological features. In addition, CCN3 localization and mechanisms of HCC progression were investigated in tissues and cell lines. Finally, correlations between CCN3 and cirrhosis were explored in patients.

**Results:**

CCN3 was primarily localized to hepatic cells of non-cancerous hepatic tissues and was associated with vascular invasion and poor prognosis in patients with HCC. CCN3 expression in non-cancerous hepatic tissues also correlated with the degree of liver fibrosis. Compared with conditioned media from wild-type LO2 cells, conditioned media from hepatic cell line LO2 activated by LX2 (aLO2-CM) induced CCN3 expression and HCC cell proliferation and metastasis. Further, aLO2-CM activated MAPK signaling and epithelial-mesenchymal transition in HCC cells. Finally, CCN3 was inversely related to cirrhosis in the prognosis of HCC and negatively regulated hepatic stellate cells (HSCs) in vitro with downregulation of α-SMA, TGF-β, and collagens.

**Conclusions:**

CCN3 was secreted from hepatic cells activated by HSCs and increased MAPK signaling, EMT, proliferation and metastasis of HCC cells. CCN3 was also inversely related to cirrhosis, regulating HSCs through a negative feedback loop.

## Background

Hepatocellular carcinoma (HCC) represents an extremely poor prognostic cancer, and more than 50% of the total number of HCC patients was occurred in China [1]. Most patients with HCC have a history of cirrhosis. As a primary risk factor for developing HCC, cirrhosis treatment is a potential strategy for preventing and treating HCC [2].

The tumor microenvironment plays a key role in HCC progression and metastasis [3]. HCC develops from chronically damaged hepatic tissue, which is surrounded by non-cancerous tissue containing stromal cells and cytokines. HCC exhibits a unique stromal signature associated with metastasis and recurrence [4]. Budhu et al. [5] previously explored the unique expression profiles of non-cancerous hepatic tissues obtained from patients with venous metastatic HCC. In the current study, we performed cDNA microarray-based gene expression profiling and found significant upregulation of CCN3, a matricellular protein encoded by the nephroblastoma overexpressed (*NOV*) gene in humans. The CCN family is a small, six-member family of cysteine-rich regulatory proteins in humans. CCN proteins function as core regulatory factors in the inflammatory microenvironment and are closely linked with HCC progression [6]. We previously reported that high CCN3 protein expression in tumor tissue is associated with low overall survival (OS) rates in patients with HCC [7]. However, CCN3 expression levels in non-cancerous hepatic tissues and the role of CCN3 in HCC progression remain unclear.

The goal of the present study was to explore expression and functional effects of CCN3 in non-cancerous hepatic tissues in correlation with HCC progression. We analyzed the localization and role of CCN3 in HCC. We found that CCN3 was secreted from hepatic cells and explored signaling pathways related to HCC malignant progression. Finally, we confirmed the antagonistic role of CCN3 on hepatic stellate cells (HSCs).

## Methods

### Patients and follow up

Clinicopathological analysis was performed on 374 HCC samples. Immunohistochemical analysis of tissue microarrays included 186 non-cancerous hepatic tissues obtained from HCC patients. Another 98 HCC samples were evaluated for CCN3 mRNA levels. Post-surgical follow up occurred until December 2013, with a median follow-up period of 63 months (range, 0–110 months). Curative resection was defined as complete resection of tumor nodules with clean post-surgical margins. Histopathological diagnosis was performed according to WHO criteria. The level of cirrhosis of liver tissue refers to the general observation of pathological specimens and the improved standard of METAVIR scoring system. The severity of cirrhosis was classified into four degree by histopathological evaluation of the liver tissue: No fibrosis, mild (portal fibrosis with few septa), moderate (numerous septa without cirrhosis), and severe (cirrhosis). The clinicopathological characteristics of patients are provided in Tables 1 and 2. Written consent was obtained from patients who received curative resection at the Liver Cancer Institute of Zhongshan Hospital of Fudan University between January 2004 and December 2006, and ethical approval was obtained from the Research Ethics Committee of Fudan University.

**Table 1:** Correlations between CCN3 expression in non-cancerous hepatic tissues and clinicopathological features of HCC patients

Variable	Patients (*N* = 186)		
	CCN3^high^	CCN3^low^	*p*
Age, y			
≥ 53	70	27	0.759
< 53	66	23	
Sex			
Male	26	7	0.418
Female	110	43	
HBsAg			
Positive	132	46	0.132
Negative	4	4	
Cirrhosis			
Yes	126	39	**0.005**
No	10	11	
AFP (μg/L)			
≥ 20	94	33	0.712
< 20	42	17	
Serum, ALT (IU/L)			
≥ 75	11	10	**0.023**
< 75	125	40	
Tumor dimension			
≥ 5 cm	26	11	0.630
< 5 cm	112	39	
No. of tumors			
Multiple	7	1	0.356*
Single	129	49	
Vascular invasion			
Yes	60	12	**0.013**
No	76	38	
Tumor encapsulation			
Complete	70	26	0.877
None	66	24	

*Fisher’s exact tests, and Chi-squared tests for all other analyses

CCN3 median values were used as cut-off points to identify subgroups (low expression and high expression groups). AFP, alpha-fetoprotein; ALT, alanine aminotransferase; HBsAg, hepatitis B surface antigen; HCC, hepatocellular carcinoma

**2:** Correlations between liver cirrhosis and clinicopathological features of HCC patients

Variable	Patients with Cirrhosis (*N* = 374)		
	yes	no	*p*
Age, y			
≥ 50	171	16	0.101
< 50	161	26	
HBsAg			
Positive	318	29	**<0.001**
Negative	14	13	
Serum, AFP (μg/L)			
≥ 20	216	20	**0.026**
< 20	116	22	
Tumor dimension			
≥ 5 cm	62	19	**<0.001**
< 5 cm	270	23	
Vascular invasion			
Yes	100	14	0.679
No	232	28	
Tumor differentiation			
I-II	253	27	0.096
III-IV	79	15	
Sex			
Male	271	35	0.823
Female	60	7	
Serum, ALT (IU/L)			
≥ 60	34	6	0.389
< 60	298	36	
No. of tumors			
Multiple	19	0	0.242*
Single	313	42	
Tumor encapsulation			
Complete	196	20	0.152
None	136	22	
Tumor recurrence			
Yes	165	16	0.162
No	167	26	

AFP, alpha-fetoprotein; HBsAg, hepatitis B surface antigen; HCV, hepatitis C virus

*Fisher’s exact test

### Cell lines and preparation of conditioned media (CM)

HCC cell line Hep3B, hepatic stellate cell line LX2, and hepatic cell line LO2 were obtained from the Liver Cancer Institute of Fudan University (Shanghai, China). All cell lines were cultured in Dulbecco’s Modified Eagle’s Media (DMEM; GIBCO, Grand Island, NY, USA) containing 10% fetal bovine serum (FBS; GIBCO) at 37 °C in a humidified incubator with 5% CO_2_.

Conditioned Media (CM) form HSC LX2 was used for treating hepatic cells LO2 for 3 days. Then LO2 cells were activated by LX2 and referred to as activated LO2 (aLO2). When we silenced endogenous CCN3 in aLO2 using lentiviral transfection, the CM from HSC LX2 was were continuously applied until the aLO2-CCN3-sh1 was successful constructed. We silenced endogenous CCN3 in aLO2 using lentiviral transfection and named these cells aLO2-CCN3-sh1. The aLO2 and aLO2-CCN3-sh1 cell lines were plated in T_75_ flasks (1 × 10^6^ cells). Media was replaced with 6 ml of fresh DMEM containing 2% FBS the next day. After 24 h, CM were centrifuged at 1000×g, and the supernatants were collected and designated aLO2-CM or aLO2-CCN3sh1-CM.

### RT-qPCR

TRIzol® reagent (Invitrogen, Carlsbad, CA, USA) was used to extract total RNA according to the manufacturer’s instructions. Reverse transcription was performed with 2 μg total RNA using the PrimeScript RT Reagent Kit (TaKaRa, Otsu, Japan). Quantitative real-time PCR was performed with SYBR Green Master Mix (TaKaRa). The primer sequences for human β-actin and CCN3 were: β-actin (forward: 5′-AAATCTGGCACCACACCTTC-3′; reverse: 5′-GGGGTGTTG AAGGTCTCAAA-3′) and CCN3 (forward: 5′-CACGGCGG TAGAGGGAGATAA-3′; reverse: 5′-TGGGCCACAGATCCACTTTTC- 3′).

### Migration and invasion assays

Migration of HCC cells was evaluated in Boyden chambers containing membranes with 8.0-μm pore size in 24-well plates (Corning, Tewksbury, MA, USA). Hep3B cells were seeded into the upper chamber of each well in serum-free DMEM (6 × 10^4^ cells per well). DMEM containing 2% FBS or aLO2-CM was added to the lower chamber of each well. After 24 h, cells on the underside of the membrane were stained with Giemsa (Sigma Chemical Company, St. Louis, MO, USA), counted, and photographed at 200× magnification. Cell invasion assays were performed similarly, except that 80 μL Matrigel (BD Biosciences, San Jose, CA, USA) was added to each well 6 h before cells were seeded onto the membrane.

### Vector construction, and transfection and lentivirus transduction

The following three CCN3 shRNAs, each in lentiviral expression plasmid PLKO.1, were used: CCCACCATCAAAGGAATATAA (Sh1), CGCACCAAGAAGTCACTCAAA (Sh2), and CACCAATAGGAACCGTCAATG (Sh3).

### Immunohistochemistry, immunoblotting, and enzyme-linked immunosorbent assays

Immunohistochemistry and immunoblotting were performed as previously described [8]. Protein concentrations were determined by the BCA Protein Assay Kit (Beyotime, Shanghai, China). Primary antibodies used for immunohistochemistry and/or immunoblotting were as follow: α-SMA, Collagen I, Collagen III, CCN3, TGF-β, p-RAF, p-MEK, p-ERK, E-cadherin, Vimentin and Actin (Abcam, Cambridge, MA, USA). Recombinant human CCN3 was purchased from Peprotech (Rocky Hill, NJ, USA). TGF-β from LX2 was quantified by ELISA (R&D Labs, Minneapolis, MN, USA). Assays were performed according to the manufacturer’s instructions in quadruplicate as previously described [9].

### Cell cycle assay

After 24 h of serum starvation, LO2 cells were grown in aLO2-CM or DMEM containing 2% FBS with recombinant human CCN3 for 24 h. Cell cycle analysis was performed by flow cytometry on a Becton Dickinson FACSCalibur™ using the Cell Cycle Detection Kit (KeyGEN, Nanjing, China) according to the manufacturer’s protocol.

### Statistical analyses

Time to recurrence rates (TRR) and OS of patients in different groups were compared by Kaplan-Meier analysis. Statistical analyses shown in Tables 1 and 2 were performed with Fisher’s exact or Chi-squared tests. Quantitative data were evaluated by *t*-tests. IBM SPSS® 20.0 for Windows was used to perform statistical analyses, with statistical significance defined as *p* < 0.05.

## Results

### High expression of CCN3 localizes primarily to hepatic cells in non-cancerous hepatic tissues and correlates with metastasis and poor prognosis in patients with HCC

**Fig. 1:**
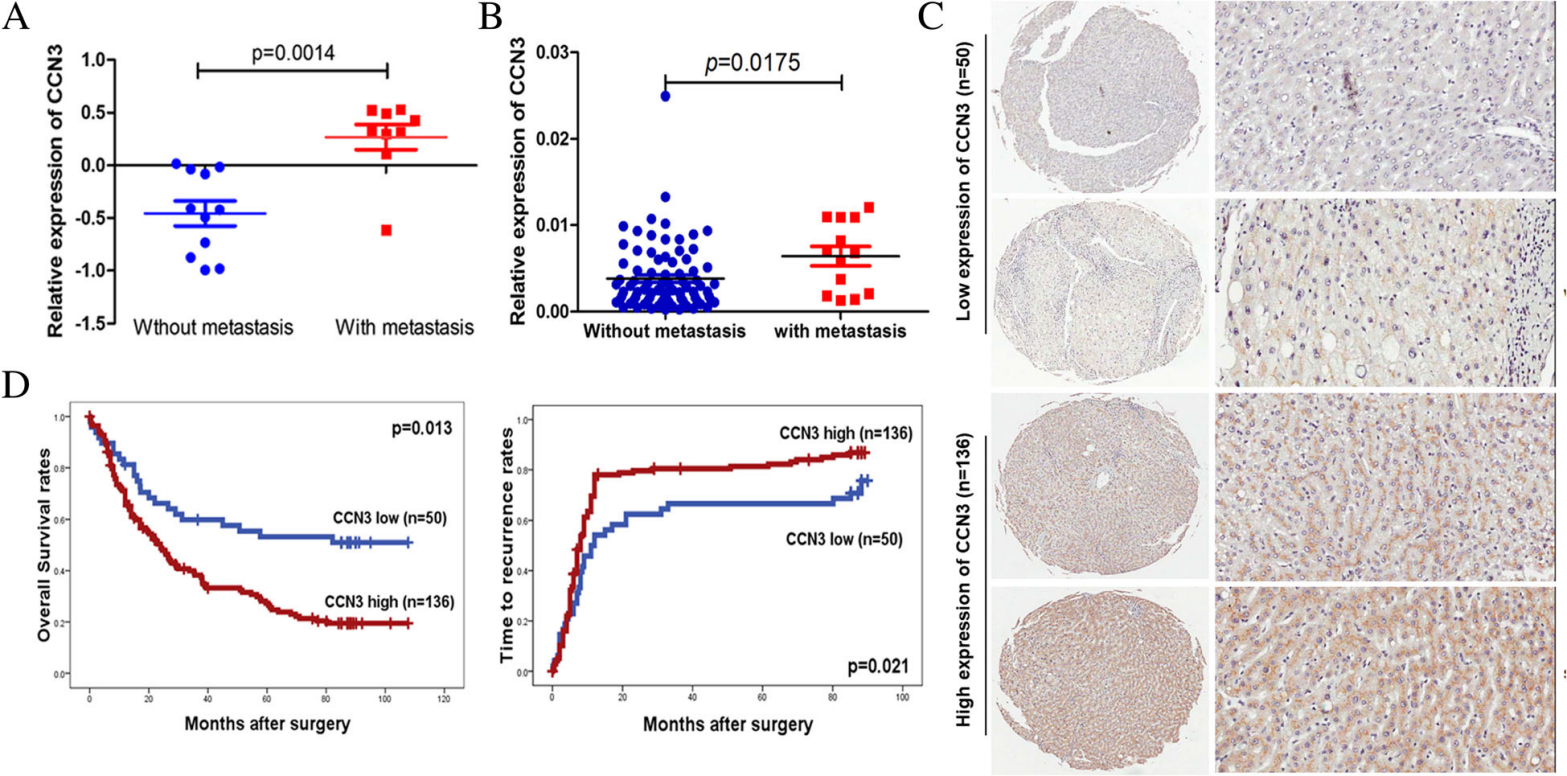
CCN3 expression in non-cancerous hepatic tissues correlates with metastasis and poor prognosis in HCC patients. CCN3 is significantly upregulated in non-cancerous hepatic tissues from patients with metastatic HCC based on cDNA microarray-based gene expression profiling (**a**). CCN3 mRNA levels are upregulated in patients with metastatic HCC (**b**). CCN3 staining localizes primarily to the cytoplasm of hepatic cells (**c**). Patients with high expression of CCN3 exhibit significantly lower OS and higher TRR (**d**)

**Fig. 2:**
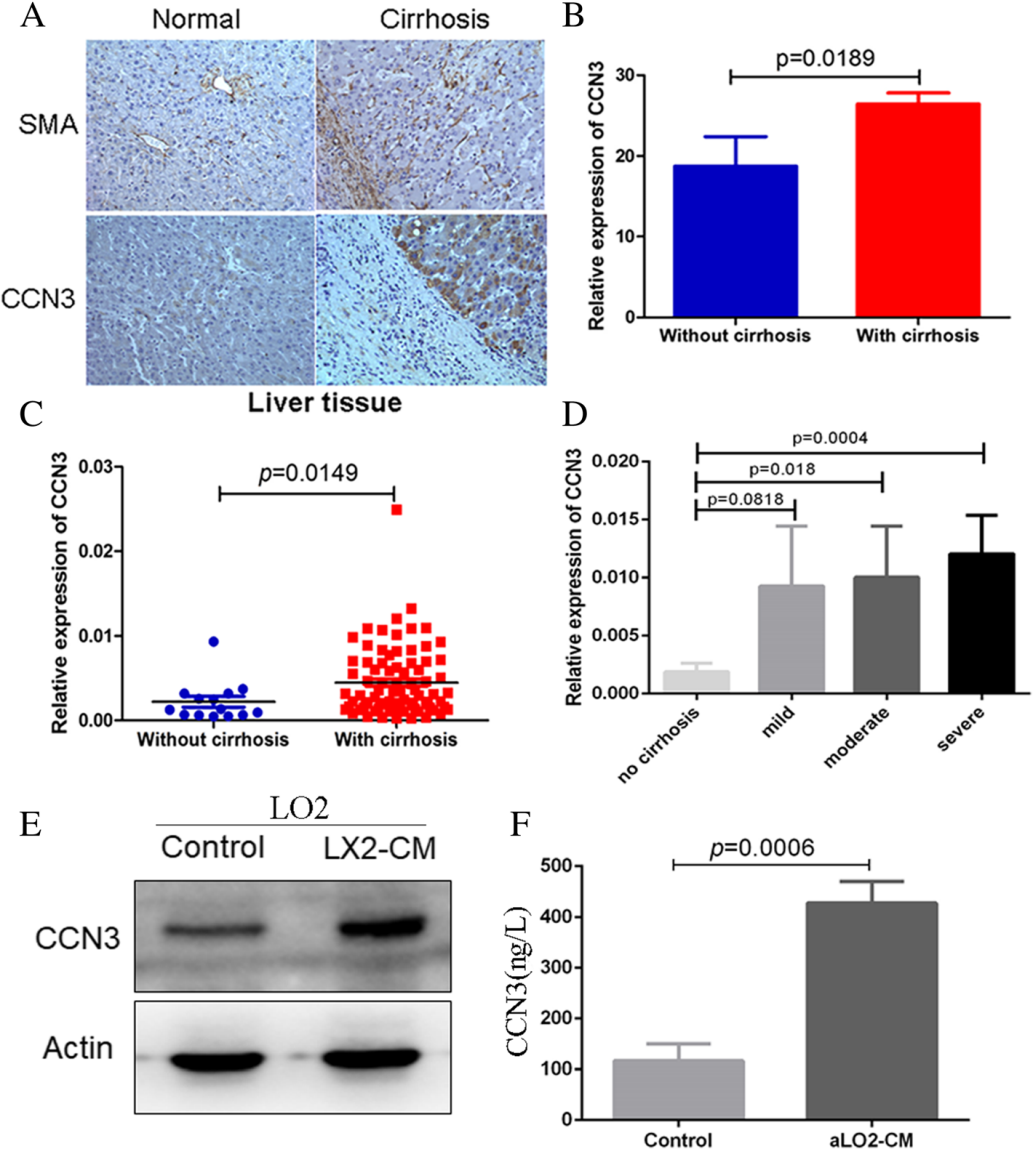
CCN3 expression in non-cancerous hepatic tissues correlates with cirrhosis. CCN3 expression in non-cancerous hepatic tissues obtained from 186 HCC patients is higher among patients with cirrhosis (**a, b**). CCN3 mRNA levels are upregulated in HCC patients with cirrhosis (**c**). CCN3 mRNA levels are increased in patients with cirrhosis according to gradient level (**d**). Western blotting shows that CCN3 levels are increased by LX2 in activated LO2 cells (**e**). ELISA shows that CCN3 expression is increased in activated LO2 cells exposed to CM compared with wild-type LO2 cells (**f**)

Budhu et al. [5] previously examined gene expression profiles in non-cancerous hepatic tissues from patients with HCC and intrahepatic venous metastasis. In the cDNA microarray-based gene expression profiles, we found that CCN3 was significantly upregulated in HCC patients with intrahepatic venous metastasis than patients without intrahepatic venous metastasis (0.27 ± 0.39 vs. -0.46 ± 0.39 *p* = 0.0014, Fig. 1a). We examined mRNA expression in 98 non-cancerous hepatic tissues from patients with HCC. CCN3 mRNA levels were upregulated in HCC patients with venous metastasis than patients without intrahepatic venous metastasis (0.0064 ± 0.004 vs. 0.0037 ± 0.0037*, p* = 0.0175, Fig. 1b). To confirm the clinical role of CCN3, we examined another 186 non-cancerous hepatic tissues by immunohistochemistry. CCN3 staining was primarily localized to hepatic cells (Fig. 1c*,* Fig. 2a). Patients with HCC and high expression of CCN3 in non-cancerous hepatic tissues exhibited significantly lower OS (*p* = 0.013) and higher TRR (*p* = 0.021) (Fig. 1d). These data demonstrate that high expression of CCN3 in non-cancerous hepatic tissues correlates with metastasis and poor prognosis in patients with HCC.

### CCN3 expression correlates with level of cirrhosis, and HSCs induce expression of CCN3 in hepatic cells

In the 186 patients with HCC presented in the previous section, Cox regression analysis revealed a positive correlation between CCN3 expression in non-cancerous hepatic tissues and cirrhosis (*p* = 0.005), serum ALT (*p* = 0.023), and vascular invasion (*p* = 0.013) (Table 1). CCN3 expression was higher in patients with cirrhosis (26.47 ± 9.81 vs. 18.73 ± 14.27 *p* = 0.0189, Fig. 2a, b), supporting a correlation between CCN3 levels in liver tissues and cirrhosis. Next, we evaluated the mRNA levels of CCN3 in 98 clinical samples. CCN3 was upregulated in non-cancerous hepatic tissues from patients with HCC and cirrhosis (0.0045 ± 0.0040 vs. 0.0022 ± 0.0023 *p* = 0.0149, Fig. 2c). Patients were then divided into four groups according to level of cirrhosis (severe, moderate, mild, and no cirrhosis). CCN3 expression was significantly upregulated in patients with severe cirrhosis compared with those who had no cirrhosis (0.012 ± 0.014 vs. 0.0019 ± 0.0026 *p* = 0.0004). Relatively low levels of CCN3 were detected in HCC patients who had moderate to mild cirrhosis compared with those who had severe cirrhosis, although this difference was not statistically significant. There was also no significant difference between the expression of CCN3 in patients with mild cirrhosis and patients without cirrhosis (0.0093 ± 0.0282 vs. 0.0019 ± 0.0026 *p* = 0.0818). CCN3 expression was significantly higher in HCC patients with moderate cirrhosis compared with those who had no cirrhosis (0.010 ± 0.021 vs. 0.0019 ± 0.0026 *p* = 0.018, Fig. 2d).

Western blotting demonstrated that CCN3 expression was increased in hepatic LO2 cells activated by HSC LX2 compared with wild-type LO2 cells (Fig. 2e). ELISA further showed that the CCN3 concentration in CM from activated LO2 (aLO2-CM) was significantly increased compared with that of wild-type LO2 (427.31 ± 42.55 ng/L vs. 116.74 ± 33.56 ng/L, *p* = 0.0006, Fig. 2f).

These findings indicate that CCN3 expression in non-cancerous hepatic tissues correlates with the gradient level of cirrhosis, and that CCN3 is significantly increased in hepatic cells activated by HSCs.

### CCN3 from hepatic cells enhances proliferation and migration of HCC in association with MAPK activation and EMT

**Fig. 3:**
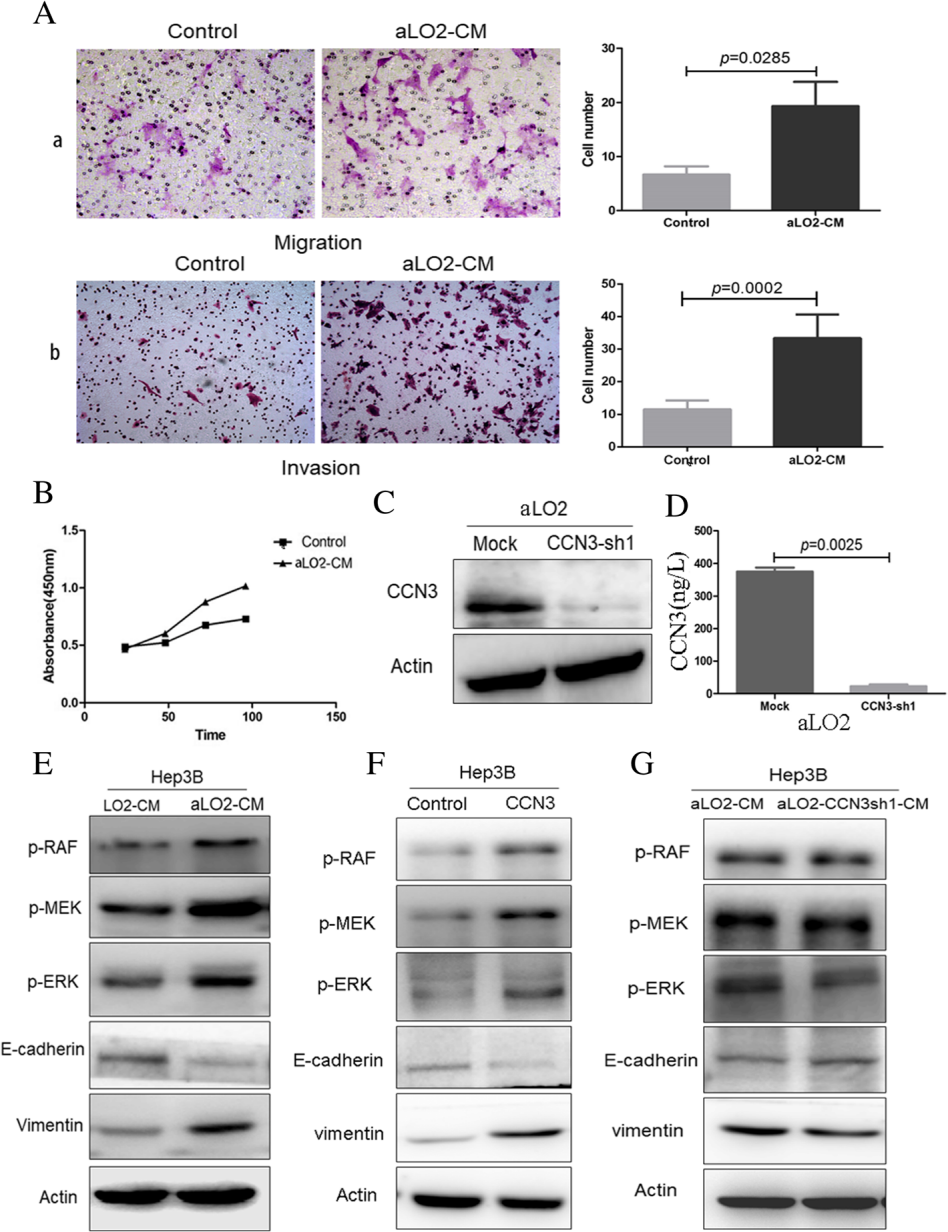
CCN3 from hepatic cells increases HCC growth and migration and induces MAPK signaling and EMT. Hep3B cells treated with LO2-CM exhibit significantly increased migration (**a, a**) and invasion (**a, b**). Hep3B proliferation is increased by LO2-CM (**b**). Endogenous CCN3 expression is significantly downregulated in aLO2 by lentiviral shRNA, as shown by Western blotting (**c**) and ELISA (**d**). MAPK signaling and EMT are induced in Hep3B treated with LO2-CM, as demonstrated by increased p-RAF, p-MEK, p-ERK, and Vimentin and reduced E-cadherin (**e**). Recombinant CCN3 induces this same trend in MAPK signaling activation and EMT (**f**). aLO2-CCN3sh1-CM inhibits MAPK signaling and EMT compared with aLO2-CM in HCC cells (**g**)

Compared with the control group, Hep3B cells exposed to aLO2-CM demonstrated significantly increased migration (5.75 ± 3.30 vs. 17.50 ± 5.79, *p* = 0.0027, Fig. 3a*, a*), invasion (11.61 ± 2.70 vs. 33.40 ± 7.27, *p* = 0.0002, Fig. 3a, b), and proliferation (Fig. 3b). Endogenous expression of CCN3 was significantly downregulated in aLO2 by lentiviral transfection (aLO2-CCN3sh1) (375 ± 21.93 ng/L vs. 22.67 ± 9.29 ng/L, *p* = 0.0025, Fig. 3c, d). Next, we investigated the effects of CM from aLO2-sh1 and wild-type cells on Hep3B signaling. Exposure of cells to aLO2-CM activated MAPK and EMT with increased p-RAF, p-MEK, p-ERK, and Vimentin and decreased E-cadherin (Fig. 3e). Similarly, recombinant CCN3 induced MAPK signaling and EMT (Fig. 3f). Conversely, CM from aLO2-CCN3-sh1 reduced MAPK signaling and inhibited EMT compared with aLO2-CM (Fig. 3g).

### CCN3 is inversely related to cirrhosis in the prognosis of HCC and reduces expression of α-SMA and TGF-β1 in HSCs

**Fig. 4:**
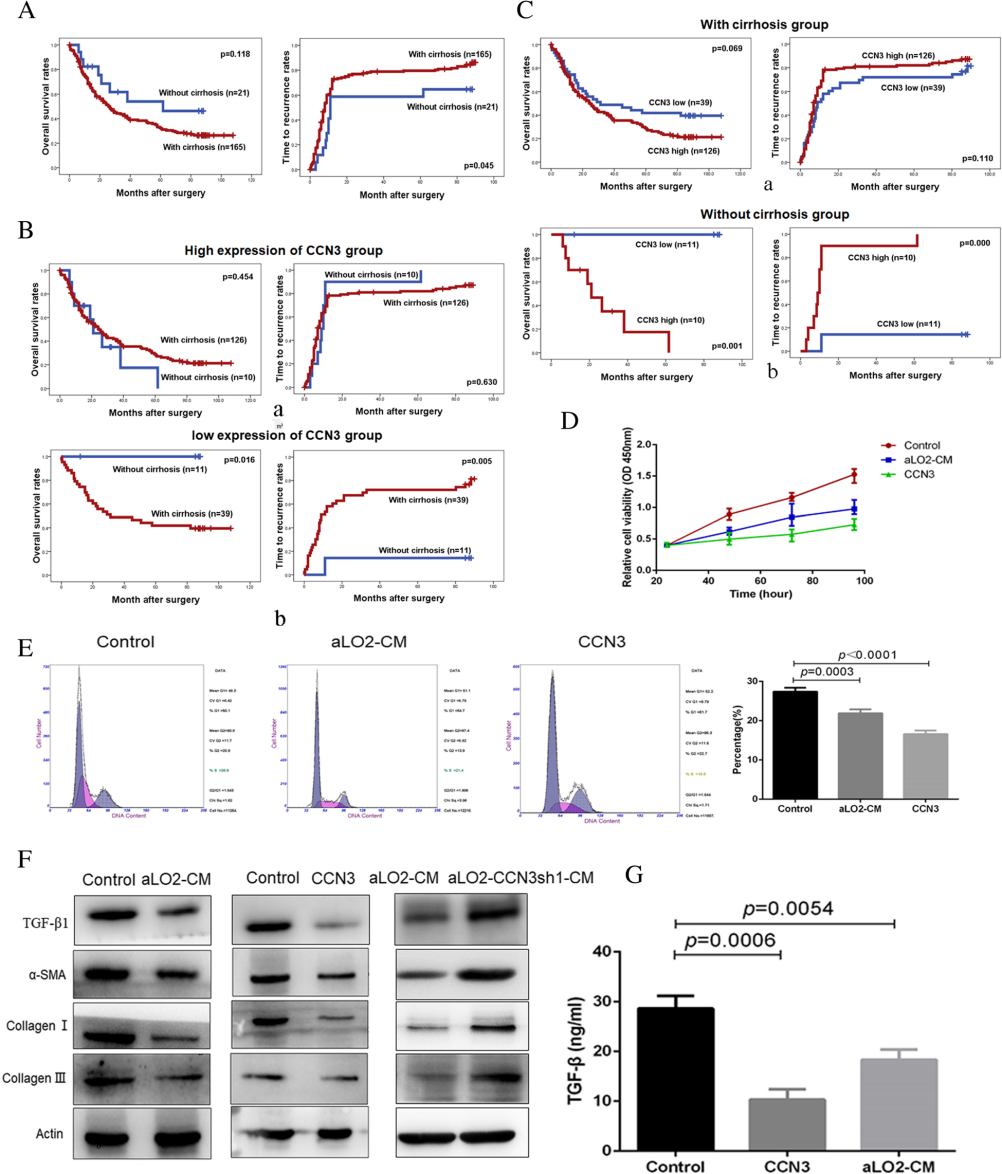
CCN3 is inversely related to cirrhosis in the prognosis of HCC and inhibits HSC proliferation. Patients with cirrhosis exhibit lower OS and higher TRR (**a**). There was no difference in OS or TRR when patients with high CCN3 expression were stratified according to presence or absence of cirrhosis (**b, a**). There was a significant difference in OS and TRR when patients with low CCN3 expression were stratified according to presence or absence of cirrhosis (**b, b**). There was no difference in OS or TRR when patients with cirrhosis were stratified according to CCN3 expression (**c, a**). There was a significant difference in OS and TRR when patients without cirrhosis were stratified according to CCN3 expression (**c, b**). aLO2-CM and CCN3 significantly inhibit proliferation of LX2 (**d**). The proportion of LX2 cells in S phase is decreased after treatment with aLO2-CM or CCN3 (**e**). LX2 cells treated with aLO2-CM or CCN3 exhibit reduced expression of TGF-β1, α-SMA, Collagen I, and Collagen III; this trend is reversed in aLO2-CCN3sh1-CM (**f**). LX2 cells treated with aLO2-CM or CCN3 exhibit significantly decreased concentrations of TGF-β1 (**g**)

In the present study, we examined the role of cirrhosis in the prognosis of 186 patients with HCC. Patients with cirrhosis exhibited higher TRR (*p* = 0.045) and lower OS (*p* = 0.118, Fig. 4a) compared with those who did not have cirrhosis. Further analysis was performed for correlations between liver cirrhosis and clinicopathological features in 374 HCC patients. Cox regression analysis demonstrated a correlation between cirrhosis and HBsAg (*p* < 0.001), AFP (*p* = 0.026), and tumor dimension (p < 0.001) (Table 2). We believe that cirrhosis alone is not a sufficient predictor of survival. To better understand the relationship between CCN3 and cirrhosis in the prognosis of HCC, we classified patients into two groups based on CCN3 expression level. Patients with high expression of CCN3 were further divided into two subgroups according to the presence or absence of cirrhosis. However, no difference in OS (*p* = 0.454) or TRR (*p* = 0.630) (Fig. 4b, *a*) was found between the two high CCN3 subgroups. By contrast, patients with low expression of CCN3 and cirrhosis exhibited a significantly lower OS (*p* = 0.016) and higher TRR (*p* = 0.005) than patients without cirrhosis (Fig. 4b, b). These data suggest that low expression of CCN3 may predict poor prognosis in patients with HCC and cirrhosis.

Patients were then divided into two groups based on the presence or absence of cirrhosis. Patients with cirrhosis were further divided into two subgroups according to CCN3 expression. There was no difference in OS (*p* = 0.454) or TRR (*p* = 0.630) between patients with high or low expression of CCN3 (Fig. 4c, *a*). However, in the subgroup without cirrhosis, patients with high expression of CCN3 exhibited significantly lower OS (*p* = 0.016) and higher TRR (*p* = 0.005) than patients with low expression of CCN3 (Fig. 4c, b). Thus, high expression of CCN3 may predict poor prognosis in patients with HCC who do not have cirrhosis.

Finally, we confirmed that CCN3 inhibits HSCs. LX2 proliferation was significantly inhibited by CM from activated LO2 or by the recombinant human CCN3 (Fig. 4d). Consistent with these data, cell cycle analyses demonstrated that the proportion of LX2 cells in S phase decreased after exposure to aLO2-CM (21.90 ± 0.99 vs. 27.38 ± 1.04 *p* = 0.0003), or exposure to CCN3 (16.58 ± 0.92 vs. 27.38 ± 1.04 *p <* 0.0001) compared with the control group (Fig. 4e). LX2 cells treated with aLO2-CM or CCN3 also exhibited downregulation of TGF-β1, α-SMA, Collagen I, and Collagen III. This trend was reversed by CM from activated aLO2-CCN3sh1 (Fig. 4f). Finally, LX2 treated with aLO2-CM (18.33 ± 2.08 vs. 28.67 ± 2.52 *p* = 0.0054) or CCN3 (10.31 ± 2.12 vs. 28.67 ± 2.52 *p* = 0.0006) exhibited significantly decreased expression of TGF-β compared with wild-type LX2 (Fig. 4g). These results demonstrate that CCN3 reduces HSC proliferation and signaling and is inversely related to cirrhosis. These data also support combined analysis of CCN3 expression and cirrhosis history to determine prognosis in patients with HCC.

## Discussion

HCC is a deadly cancer and is usually accompanied by cirrhosis due to chronic inflammation and necrotic hepatic tissue [10]. Numerous studies indicate that HCC progression is greatly influenced by the tumor microenvironment [11, 12]. The cirrhosis microenvironment supports the malignant progression of HCC [13, 14]. Understanding the role of cirrhosis in HCC progression may help identify new therapies for a more personalized medical approach [15, 16].

HSCs are a key cell type in cirrhotic tissue and play an important role in hepatic fibrosis [17]. Activated HSCs promote HCC progression primarily through paracrine effects. We previously showed that HSCs interact with hepatoma cells via secretion of cytokines, which modulate the malignant changes of HCC [18, 19]. In this study, we confirmed that patients with cirrhosis have a higher TRR and lower OS, although the differences were not significant. Therefore, identification of a molecular marker in HCC with a cirrhosis microenvironment may improve understanding of the relationship between cirrhosis and HCC. Budhu et al. [5] demonstrated that the *NOV* gene, which encodes the secreted protein CCN3 in humans, is significantly upregulated in non-cancerous hepatic tissues from patients with HCC and intrahepatic venous metastases. In the current study, tissue microarrays showed that CCN3 localized primarily to the hepatic cells of non-cancerous hepatic tissues. CCN3 was significantly upregulated in HCC patients with vascular invasion and poor prognosis. Tissue microarrays also demonstrated that increased expression of CCN3 in hepatic cells correlated with the level of cirrhosis. In vitro analyses showed paracrine effects of CCN3 from hepatic cells; these effects were significantly increased in hepatic cells activated by HSCs. Therefore, CCN3 paracrine effects from hepatic cells may be closely related to cirrhosis and may play an important role in HCC progression.

The CCN family includes six protein members. Similar to other family members, CCN3 is composed of the following four functional modules, IGFBP, VWC, TSP1, and CT. CCN3 functions as a localized multitasking signal integrator in the microenvironment, binding with multiple receptors involved in the regulation of cell proliferation, chemotaxis, angiogenesis, and adhesion [20]. CCN3 was overexpressed in metastatic melanoma compared with the primary tumor and was associated with higher metastatic potential of melanoma cells [21]. Previously, our group showed that CCN3 is a potential therapeutic target that may affect upregulation of Ostopontin (OPN)and coagulation factors, enhancing the stemness and blood coagulation microenvironment of HCC tissue [7]. We also found that CCN3 orchestrated the stroma-derived resistance to chemotherapy in HCC. In the present study, we demonstrated that treatment of HCC cell line Hep3B with aLO2-CM, which has high CCN3 levels, induced migration and proliferation with upregulation of p-RAF, p-MEK, p-ERK, and Vimentin and downregulation of E-cadherin. These results indicate that hepatic cells promote the malignant progression of HCC via MAPK signaling and EMT.

**Fig. 5:**
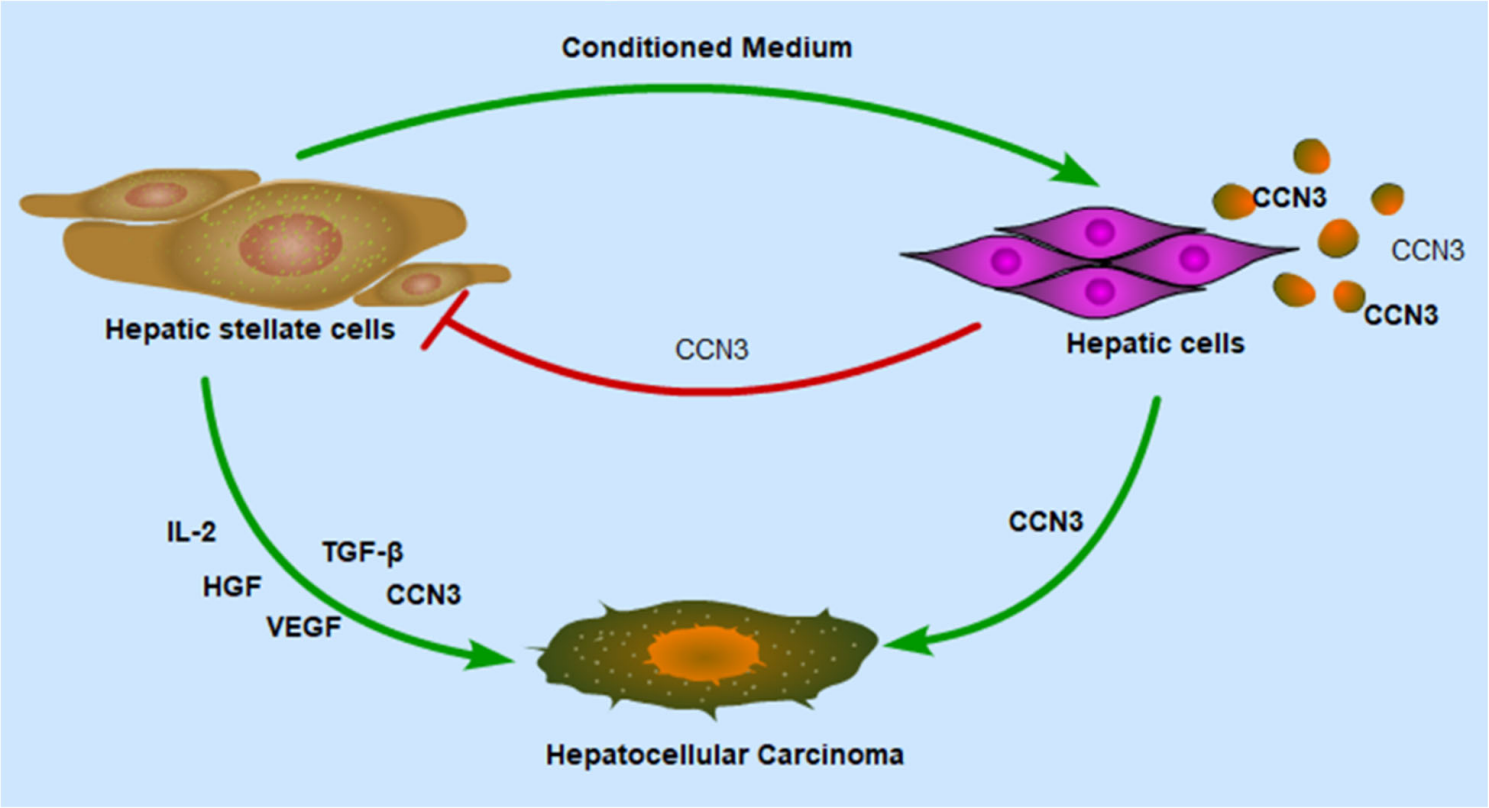
The role of hepatic cells and HSCs in the malignant progression of HCC. HSCs induce secretion of CCN3, which in turn inhibits proliferation of HSCs. HSCs and activated hepatic cells exert paracrine effects to promote HCC progression

The CCN family of proteins also participate in the assembly of the extracellular matrix [22]. In the present study, we examined the expression of CCN3 in hepatic tissue microarrays. High expression of CCN3 was localized to hepatic cells and associated with more severe cirrhosis. In addition, CCN3 was significantly increased in hepatic cell line LO2 after treatment with LX2-CM. Andrew et al. [23] reported that CCN3 is a potential anti-fibrotic treatment. We further showed that CCN3 was inversely related to cirrhosis in the prognosis of HCC and inhibited expression of α-SMA and TGF-β1 in HSCs. In patients without cirrhosis, high CCN3 expression correlated with lower OS and higher TRR compared with those who had low CCN3 expression. In patients with low CCN3 expression, patients with cirrhosis had significantly worse prognosis than patients without cirrhosis. We also concluded that CCN3 was inversely related to cirrhosis in the prognosis of HCC and acted in a negative feedback loop in HSCs (Fig. 5).

Although cirrhosis promoting tumors progression is very clearly, while cirrhosis was insufficient factor alone to predict prognosis of patients with HCC, cause of many cytokines such as CCN3 may influence the relationship between cirrhosis and HCC progression. Maybe that is why anti-fibrosis is not an very effect way to anti-HCC. Further studies will focus on the relationship between HCC and cirrhosis to identify a comprehensive treatment strategy.

## Conclusions

From our experimental results and our review of the literature, we propose the following conclusions. (1) HCC progression is profoundly influenced by the tumor microenvironment. (2) Cirrhosis and CCN3 in hepatic cells are associated with a malignant phenotype and poor prognosis in HCC. (3) Increased paracrine effects of CCN3 from hepatic cells was induced by HSCs, enhancing the growth and migration of HCC with activation of MAPK signaling and EMT. (4) CCN3 reduced expression of TGF-β1 in HSCs, acting in a negative feedback loop in HSCs.

## Abbreviations

CM: Conditioned media
DMEM: Dulbecco’s Modified Eagle’s Media
EMT: Epithelial-mesenchymal transition
FBS: Fetal bovine serum
HBV: Hepatitis B virus
HCC: Hepatocellular carcinoma
HSCs: Hepatic stellate cells
NOV: Nephroblastoma overexpressed protein
OS: Overall survival rates
PCR: Polymerase chain reaction
TRR: Time to recurrence rates
